# Jordan’s Pandemic Influenza Preparedness (PIP): A Reflection on COVID-19 Response

**DOI:** 10.3390/ijerph19127200

**Published:** 2022-06-12

**Authors:** Khalid A. Kheirallah, Mohammed Al-Nusair, Shahed Aljabeiti, Nadir Sheikali, Abdallah Alzoubi, Jomana W. Alsulaiman, Abdel-Hameed Al-Mistarehi, Hamed Alzoubi, Ayman Ahmad Bani Mousa, Mohammed Z. Allouh

**Affiliations:** 1 Faculty of Medicine, Jordan University of Science and Technology, Irbid 22110, Jordan; mohammed.alnusair99@gmail.com (M.A.-N.); smaljabeiti188@med.just.edu.jo (S.A.); sheikhalinader@gmail.com (N.S.); aaalzoubi28@just.edu.jo (A.A.); awalmistarehi18@med.just.edu.jo (A.-H.A.-M.); 2 College of Medicine, Ajman University, Ajman P.O. Box 346, United Arab Emirates; 3 Faculty of Medicine, Yarmouk University, Irbid 21163, Jordan; jomana.a@yu.edu.jo; 4 Faculty of Medicine, Mutah University, Karak 61710, Jordan; dr_alzoubi@yahoo.com; 5 Jordan Ministry of Health, Amman 11118, Jordan; dr.aymanbm@gmail.com; 6 College of Medicine and Health Sciences, United Arab Emirates University, Al Ain P.O. Box 15551, United Arab Emirates

**Keywords:** COVID-19, SARS-CoV-2, influenza, pandemic, PIP, preparedness, response, national inventory, Jordan

## Abstract

The COVID-19 pandemic made it clear to the world that better preparedness for future pandemics is paramount. This study aims to explore how the 2018 Jordan’s Pandemic Influenza Preparedness (PIP) assessment plan (conducted utilizing a standardized tool of the CDC National Inventory of Core Capabilities for Pandemic Influenza Preparedness and Response) reflected on the initial COVID-19 response. A qualitative, single intrinsic case study design, utilizing interpretivist approach, was utilized to interview subject-matter experts and explore the potential reflection of PIP assessment on COVID-19 response. Utilizing a mini-Delphi approach, the interviews aimed at generating an in-depth understanding of how the Jordan’s PIP risk assessment reflects on the country’s response to COVID-19. The following 12 core capabilities, along with their reflections on COVID-19, were assessed: country planning, research and use of findings, communications, epidemiologic capability, laboratory capability, routine influenza surveillance, national respiratory disease surveillance, outbreak response, resources for containment, community-based interventions to prevent the spread of influenza, infection control (IC), and health sector pandemic response. Jordan’s experience and preparedness for influenza may have served as a crucial guide to establishing success in COVID-19 control and mitigation. Surveillance, outbreak, and research activities were very well established in Jordan’s PIP, whereas surge capacity in human capital and health facility were identified as two high-risk areas. However, the limitation in these two areas was met during the COVID-19 response. Still, human capital suffered fatigue, and there was an evident lack of laboratory testing plans when COVID-19 cases increased. Jordan’s experience with PIP may have served as a guide for establishing successful COVID-19 control and mitigation. The established PIP principles, systems, and capacities seem to have reflected well on fighting against COVID-19 in terms of more efficient utilization of available surveillance, laboratory, outbreak management, and risk communications. This reflection facilitated a better mitigation and control of COVID-19.

## 1. Introduction

One of the greatest disasters the world has ever experienced is the coronavirus disease-19 (COVID-19) pandemic (the disease caused by severe acute respiratory syndrome coronavirus-2 (SARS-CoV-2)), which has had devastating effects on the global healthcare system. Immense pressure from the pandemic on the world’s healthcare systems threatened their collapse, and governments were forced to take drastic measures to ‘flatten the curve’, imposing border shutdowns, travel restrictions, and strict lockdowns that saw many businesses close indefinitely, leading to many people losing their jobs [1]. This was the case in Jordan, where the abruptness of the first wave of COVID-19 (March 2020 to April 2020) led the government to declare a state of emergency and enforce national defense law, leading to a complete socio-economic lockdown. These strict actions helped produce a small, short-lasting first epidemic wave. However, the economic and social repercussions of such measures soon made easing them inescapable. Accordingly, the second and third waves of new COVID-19 cases and deaths (spanning from September 2020 to January 2021 and late January 2021 to May 2021, respectively) were much more significant in magnitude than the first one. By the end of May 2022, the country reported about 1.7 million COVID-19 cases, out of its 10.3 million total population (159,321 cases per 1 million population), more than 14,000 deaths (1351 deaths per 1 million population), about 17 million tests, and more than 4.5 million fully vaccinated individuals (42% of the total population) [2]. 

The COVID-19 pandemic made it clear that better preparedness for future pandemics is paramount. Nevertheless, this pandemic was hardly our first. From 1918 to 1920, the Spanish Flu killed approximately 50 million people. In 1957-58 H2N2 (1 m deaths), 1968 H3N2 (1 m deaths), and in 2009, H1N1, aka swine flu, was responsible for approximately 284,000 deaths [3]. Public health organizations were acutely aware of the need to better prepare for future outbreaks or pandemics. They have taken several steps in that direction, particularly regarding influenza pandemics, as many countries have developed, and continue to implement, national pandemic influenza preparedness (PIP) plans. However, reviews of the International Health Regulations (IHR) functioning concerning the 2009 pandemic of influenza A/H1N1 concluded that the world is not prepared for the next severe pandemic [4].

In 2011, the World Health Organization’s (WHO) Member States unanimously adopted the PIP Framework, which aims to improve the sharing of surveillance data on influenza viruses with pandemic potential and increase the access of developing countries to vaccines and other life-saving products during a pandemic [5]. Interestingly, the PIP framework’s annual progress report for the year 2020 provided evidence that measures taken to strengthen the preparedness and response of nations to influenza pandemics were helpful in the COVID-19 pandemic in a variety of ways, including National Influenza Centers (NICs) serving as national reference laboratories, integrating the reporting of COVID-19 community transmission surveillance data into influenza platforms and the framework of support established for the PIP being used to organize webinars with regulators and regional officers to expedite the approval of COVID-19 vaccines, among others [6]. Hence, the established systems and tools developed for influenza pandemics can be utilized and generalized to other viral pandemics, including coronavirus. Even more generalizable systems generated to combat public health disasters or other non-health-related disasters may be of assistance during a pandemic. For instance, Jordan’s National Center for Security and Crises Management (NCSCM), a government-led team of multidisciplinary subject-matter experts set up in 2015 to form comprehensive, strategic plans for national emergencies and disasters, health-related or otherwise, played an imperative role in Jordan’s response to COVID-19.

The WHO’s Eastern Mediterranean Region (EMR) is at particular risk for pandemics owing to many factors, including the protracted conflicts and turmoil that cause internal displacement of persons and emigration to other regions, as well as the mass gathering and close contact that occurs during the annual Islamic pilgrimage (Hajj) in Saudi Arabia. The region is also located amidst four of the eight global migratory bird flight paths, making it a hot spot for zoonotic transmission of avian influenza viruses [7]. Furthermore, studies have shown that the EMR countries’ preparedness for possible pandemics or epidemics is variable and generally lagging and research on the subject is limited [7,8]. This was obvious throughout the COVID-19 pandemic by illustrating the importance of a more significant commitment to research on pandemic preparedness [9]. It should also be stressed that for any plan aiming to enhance epidemic and pandemic preparedness in the EMR to be truly effective, a One Health approach must be considered, as the region is already known to be at risk of increased transmission of zoonotic diseases, with a well-documented history of such diseases (avian influenza A(H5N1), pandemic H1N1/2009 virus and Middle East respiratory syndrome coronavirus (MERS-CoV)) [10]. 

In 2016, the Joint External Evaluation (JEE) of International Health Regulations (IHR) core capacities reported Jordan’s “Developed Capacity” against health security risks. Still, the report identified areas with limited, or no, capacities. These included national laboratory system; reporting; preparedness; emergency response and operations; risk communication; and points of entry. The report also indicated that, overall, the health security risks in Jordan remain high due to threats of communicable diseases and pandemics [11]. As such, Jordan’s need to implement comprehensive preparedness and response actions against potential pandemics is still a concern and more investigation is needed in this regard.

Jordan has direct support from the WHO’s Regional Office to strengthen its preparedness for pandemic influenza as part of the PIP Framework. In 2018, it performed a thorough assessment of its PIP using a standardized tool developed by the U.S. Centers for Disease Control and Prevention (CDC), the National Inventory of Core Capabilities for Pandemic Influenza Preparedness and Response (national inventory tool), to provide accurate, comparable information on levels of preparedness and response across countries over time based on twelve core capabilities [4]. An overview of the results of each core capability assessed is provided in Table 1. In short, immediate attention is required for building surge capacity for health sector pandemic response, followed by communications plan (for messaging, dissemination and staffing), research priorities, laboratory network, cross-notification for respiratory diseases surveillance and reporting, exercising the management and distribution of resources for containment, and community-based interventions to prevent the spread of influenza (social distancing, critical infrastructure, and district level plans). 

Given that Jordan was hard hit by COVID-19, yet has performed well in the overall PIP risk assessment, it is then necessary to explore the effect of pandemic influenza preparedness and response in line with COVID-19 response. In theory, pandemic influenza preparedness and response shall serve as a road map for COVID-19 response, or, at least, ensure pandemic influenza preparedness and response are translated into, and reflected on, proper COVID-19 response. In the current case study, we explored how the PIP risk assessment reflected on the COVID-19 response in Jordan during the early stages of the pandemic. In specific, how each of the 12 core capability indicators was implemented within the scope of the COVID-19 response. Reflecting on the PIP risk assessment for COVID-19 response should assist Jordan and the region in preparing for future health threats of regional or international interest, including the next pandemic. This is critical given the lack of such research activities within developing or fragile conflict-prone countries in the region. 

## 2. Materials and Methods

Originally, in 2018, the National Inventory of Core Capabilities for Pandemic Influenza Preparedness and Response (PIP National Inventory) was used to assess coverage, quality, and timeliness indicators in twelve core capabilities (domains) of pandemic influenza preparedness and response. These twelve core domains included country planning, research and use of findings, communications, epidemiologic capability, laboratory capability, routine influenza surveillance, national respiratory disease surveillance and reporting, outbreak response, resources for containment, community-based interventions to prevent the spread of influenza, infection control, and health sector pandemic response. For each of the twelve domains, there are four indicators. Each indicator is divided into four performance levels, ranging from no or limited capability (coded as 0) to fully capable (coded as 3). These capabilities and indicators are limited to those related to human health (3). Detailed notes for each indicator are provided to clarify and further operationalize its contents. These notes include the primary question(s) to be asked for each capability, definitions of terms relevant to the indicators, examples of sources of information or documentation relevant to the indicators, and references to support the indicators as essential components of preparedness and response. Jordan’s PIP National Inventory, conducted in 2018, collected data using structured interviews with subject-matter experts utilizing a standardized data collection matrix developed by the CDC. Details about the tool are reported elsewhere [4]. The results of Jordan’s PIP were used as a guiding frame for the current case study.

This case study (conducted September–November 2020) explored how the PIP risk assessment (completed in 2018) reflected on the COVID-19 response in Jordan during the early stages of the pandemic (March 2020 to March 2021). In specific, the study explored how each of the 12 core capability indicators was implemented within the scope of the COVID-19 response. The theoretical framework is that Jordan’s performance in the PIP risk assessment, completed in 2018, should be reflected in its response to COVID-19. That is, how better PIP infrastructure reflects on a more efficient utilization of available PIP resources (surveillance, laboratory, outbreak management, risk communications, clinical surge capacity, and product deployment) to mitigate and control COVID-19. This highlights the collateral benefit of PIP for other emergencies, especially when considering that PIP may have been helpful in refining the COVID-19 response by helping to characterize key epidemiological features, understand the spread, severity, and spectrum of disease, identify the impact on communities and guide the use of countermeasures such as case isolation and contact tracing [6]. 

A single intrinsic case study design, utilizing interpretivist approach, was used to generate an in-depth understanding of how the PIP risk assessment for Jordan, conducted in 2018, reflects on the country’s response to COVID-19 during the early stages of the pandemic. A mini-Delphi approach was conducted by providing participants (N = 15 subject-matter experts) with the results for each PIP indicator and then asking them to find consensus on how each of the 12 core capability indicators was implemented within the scope of the COVID-19 response. The results of each of the 2018 PIP assessment indicators (Table 2) served as the base for questions asked to participants. Each subject-matter expert was asked to reflect on how the indicator-specific results reflected on the COVID-19 response. We were particularly interested in identifying and understanding subject-matter experts’ experiences, expectations, and perceptions related to the COVID-19 response under a total of 12 core capability indicators. Moreover, we were interested in assessing how Jordan responded to COVID-19 under a standardized instrument used to assess influenza preparedness. 

Around mid-September 2020, subject-matter experts and public health professionals working on combating COVID-19 were asked to review the 2018 Jordan’s PIP report and indicators and then interviewed asking them to reflect on Jordan’s COVID-19 response related to their area(s) of expertise. A total of 25 interviews were completed. Experiences, expectations, and perceptions related to COVID-19 response under a total of 12 core capability indicators were recorded and further discussed with participants. Participants’ reflections were then summarized in line with each core capability. Interviews were conducted using the Microsoft Teams software application given the COVID-19 situation in Jordan. Each interview extended for about 2 h and some participants were scheduled for more than one interview. The English language was used for all interviews. Notes were taken by a note taker and a summary of each interview was presented to the participant for approval as per the request of participants. After each interview, the research team were briefed on the interview results and was asked to emphasize on how each participant shared their response in line with the study objectives. Once finalized, notes were shared with participants for review and approval to eliminate any potential confusion, error, or misunderstandings of reported data. Edits were reported and discussed with the research team. Training of the interviewer and note taker was also completed before the study. The research team also corroborated participants’ responses utilizing available literature, scientific online resources, and Ministry of Health hotline and personnel. As well, contradictory answers were clarified with participants, and a final decision in this regard was approved by the research team. 

IRB was approved by the Jordan Ministry of Health. Participants were identified by the research team, invited to participate in the study, and then verbally consented. Study objectives were shared with participants and their anonymous responses and interview privacy were assured. No personal data was collected, and pseudo names were used when taking, reviewing, and preparing the notes. Privacy was also assured and only the researcher and note taker were allowed to attend the online interview. Interviews were conducted in a private room and all notes were secured in a private cabinet until properly disposed of after the study. None of the interviews were recorded but the note taker and researcher conducting the interview prepared meeting notes, which summarized participants’ reflections, and shared them with the participant, anonymously, for final approval. The researcher was allowed to summarize the main points and not to use quotations given the political climate around COVID-19 at the time of the interviews. 

## 3. Results

Results of the 2018 PIP (National Inventory) assessments are presented in Table 2. The assessment showed that Jordan has a well-established Pandemic Influenza Preparedness and Response infrastructure. While surveillance, outbreak, and research activities were very well established, the results identified weak points in social mobilization and health sector capacity building in terms of human capital and physical facilities and equipment. 

### 3.1. Core Capability 1: Country Planning 

The Jordan MOH published its Pandemic Influenza Preparedness and Response Plan in 2017, which is based on WHO pandemic phases and has been provided financial resources by WHO based on a needs assessment from MOH. The plan served to define the decision-making structure in the event of a pandemic. During initiation, parts of the plan were tested by the MOH Operation Center through dialogue with stakeholders. However, in order to properly assess and improve the adequacy/clarity of plans, policies, and procedures, the entire plan should be tested using at least one tabletop exercise that engages all relevant stakeholders in the process. The plan is currently only available in English, and Arabic translation was not unavailable. The plan clearly stated that available surveillance data, new research findings, revised guidance documents or international recommendations, lessons learned from exercises or simulations, and additional experience in outbreak investigation should be further utilized to strengthen pandemic preparedness and response in Jordan. 

Jordan used the available pandemic influenza preparedness and response plan (PIPRP) to activate a response to COVID-19 and, in theory, used all available influenza infrastructure and assets to respond to the pandemic. A plan introduced a national response protocol and allowed for testing of different components of the response structure. The PIPRP offered a clear definition of roles and responsibilities in responding to potential pandemics, such as COVID-19. The Plan may have helped guide successful response at the national level, where evidence was available from completed exercises or simulations. The usefulness of the PIP plans in responding to COVID-19 was also experienced in other parts of the world. For example, in Europe, Armenia’s COVID-19 Preparedness and Response Plan was developed early in the pandemic and was primarily based on national guidelines and protocols previously established with PIP support. In the Philippines, tabletop exercises conducted by an interagency contingency planning workshop with participants from many departments, including health and agriculture, in November 2019 to test the Philippines’ PIP plan provided lessons and recommendations and proved invaluable in informing the national approach to COVID-19 [6]. While Jordan’s PIPRP may not be optimal or comprehensive, it is still an advantage when considering the national response to a disease with global impact. 

### 3.2. Core Capability 2: Research and Use of Findings for Pandemic Influenza Preparedness

Jordan scored 1.7, out of 3, in Research and Use of Findings for Pandemic Influenza Preparedness, with the lowest score reflected in identifying research priorities. All other attributes, however, were above average. The utilization of surveillance data to inform decisions for PIP is evident, but well-defined research priorities for PIP are not established, limiting the utility of such data concerning decision making. Moreover, despite the participation and scientific engagement of MOH staff in international/domestic influenza conferences, there is minimal sharing of scientific evidence from Jordan abroad. 

During COVID-19, Jordan established a fair epidemiological and virological database. However, it presented little research on local evidence-based practices in this regard. The Western experience may not be reliable given the cultural and socio-demographic differences, so better local research is needed. The One Health approach may assist here as a collaboration between different surveillance systems will force electronic surveillance to detect an epidemic or an increase in zoonotic disease quickly. One Health should also be utilized to determine research priorities not only on PIP but also on other zoonotic diseases of interest to ensure a national response better equipped with tools to identify zoonotic disease at an early stage before it affects human life.

Collaboration between MOH and other relevant stakeholders is observed, and the One Health approach may be better established in Jordan in contrast to its neighbors, with documented evidence of collaboration between different research, health, and governing bodies involved in the human–animal–environment interface [10]. Such collaboration has been an asset for combating COVID-19 in Jordan, facilitating swift response at the central level and the peripheries [12].

### 3.3. Core Capability 3: Communication

In 2019, emergency risk communication (ERC) capacity-building training was conducted in Jordan as part of the PIP plan. Accordingly, Jordan updated the communication action plan that serves as an operational document for Influenza pandemic-related communications. A national spokesperson oversees the dissemination of pandemic data and updates tailored to target audiences using formal and informal communication channels and Arabic translations when needed. Nevertheless, communication and messaging remain centralized within MOH in Amman, the capital of Jordan, with only one national spokesperson in charge of communication. Development and implementation of communication and dissemination strategies at the sub-national level are recommended.

The COVID-19-related infodemic was a significant concern during the epidemic. Social media outlets and the channels they provide for widespread dissemination of information by any user introduce a novel political dimension to the COVID-19 epidemic, where opposing political views encouraged the spread of mis- and dis-information. Having a well-established communication plan provides the public with clear and concise messages about the pandemic. For instance, public health officials, who were viewed as having sufficient scientific background and no obvious political ties, were chosen to present pandemic situation reports and updates to the public. This scientific presentation of information, along with other measures taken by the government, including ensuring transparency of actions during the lockdown, aided in gaining public trust in the messages distributed by the state at every stage of the pandemic response, despite previous distrust in the system and a below-average PIP score. 

### 3.4. Core Capability 4: Epidemiologic Capability

According to the PIP assessment, the MOH had adequate staffing (more than 40 ‘practicing’ public health epidemiologists appointed within the MOH central and peripheral offices) and resources for timely monitoring and making recommendations on population health status and disparities, and acute incidents at the sub-national level. The MOH was also reported to have advanced public health/epidemiology training programs with dedicated resources, accreditation, and annual cohorts of graduates in line with the national and regional needs. 

Jordan has a centralized health system model that depends on the MOH for resource allocation, priority setting, leadership, staff, budget, and equipment, encouraging public health professionals to move centrally (to the central offices in the capital, Amman) after earning an advanced epidemiology degree or certificate. The reports recommend that the MOH should focus more on capacity building of epidemiologists at the local level and encourage decentralization of decision making, especially in rural areas at higher risk of influenza outbreaks. 

In responding to COVID-19, the MOH utilized its army of epidemiologists for case identification, investigation, and contact tracing. These efforts were supplemented by the Royal Medical Services and private enterprises to meet central and peripheral needs. Epidemiological investigations and NPI measures, such as lockdowns, were effective during the initial stage of the COVID-19 pandemic as the epidemiologic curve was successfully flattened for about three months until August 2020 [13]. In addition, there was evidence of the decentralization of government decision making during the COVID-19 response, as some cities were cleared for a partial opening while others were not. Within this context, epidemiological leaderships were established at local levels and successfully conducted surveillance activities. 

### 3.5. Core Capability 5: Laboratory Capability

The MOH Central Public Health Laboratory Directorate, a bio-safety level 2 laboratory (basic bio-safety—primary health services, diagnostic services, and research) that conducts routine testing of influenza specimens and participates in the WHO External Quality Assurance Project (EQAP), uses real-time PCR techniques to detect influenza A, avian influenza (H7N9, H5N1), swine influenza (H1N1, H3N2, H1N2), and influenza B viruses. Regular samples are collected from four severe acute respiratory infections (SARI) and three influenza-like illnesses (ILI) sites. Samples are then transported to the Directorate, located in Amman, and within 48 h of laboratory confirmation, the Directorate actively reports and shares results with the focal point(s), who then share results with the WHO through the IHR. As for unlisted virus samples, they are sent to referral labs, such as CDC, after the MOH alerts the Emergency Operations Centre (EOC) of detecting a novel virus. Of note, laboratory services related to influenza are centrally located in the capital Amman with limited testing resources in peripheral sites. This could be disadvantageous during a pandemic as the workload on the Directorate may become overwhelming. Moreover, routine SOPs related to influenza laboratory activities are not adequately developed, such as safety and security, transportation of specimens, testing validation, and reporting. 

Within the scope of COVID-19, the Laboratory Directorate was initially sufficient to test COVID-19 samples (from cases and contacts) using real-time PCR, as the number of cases between March and July was not high enough to stress the central lab in Amman. However, with the surge in cases during the second wave, regional laboratories were tasked with meeting the high testing demand. Private lab systems and drive-through services were sufficient to meet the surges of testing. Availability of SOPs to conduct COVID-19 sample testing was noted during the pandemic, and training according to international standards was provided. Timely reporting of COVID-19 test results and using an SMS messaging system to communicate the results were an advantage supported by the MOH and further facilitated the timely reporting of daily newly diagnosed cases and positivity rates. The ability to utilize regional and peripheral laboratory services, including private ones, to meet the high demand in the central laboratories was helpful in meeting demand, especially as the central Directorate became overwhelmed. Establishing testing during unexpected surges in numbers and ensuring timely reporting of results is critical. The presence of well-established laboratory capacities in Jordan undoubtedly aided in mitigating the COVID-19 response. This was in line with other world experiences, such as the South-East Asia Region (SEAR), where all 11 SEAR countries had built the capacity to accurately and reliably detect influenza viruses through real-time PCR, as recognized by 2019 WHO EQAP, which proved instrumental in the providing the basis for COVID-19 testing in the region [6].

### 3.6. Core Capability 6: Routine Influenza Surveillance

Jordan’s PIP reports up-to-date integrated virological and epidemiologic surveillance systems from four SARI and three ILI sites, both of which use clear case definitions that are age-appropriate and in accordance with WHO standards. The geographic distribution of sentinel networks adequately represents the various geographic and climatic regions and population groups in the country. Live (online) data entry systems enhance the timeliness of reporting and provide live surveillance updates. Distribution and publication of MOH surveillance data, including sentinel sites’ virologic and epidemiologic data, have been documented as regular bulletins, newsletters, email correspondence, websites, and scientific journals [14]. However, the timeliness of data sharing remains an issue, especially when considering a One Health approach as an accepted pattern of communicating data-related messages between stakeholders. This is to save time and allow for direct communication between the ministries of health, agriculture, and the environment, but this channel communication is not well established. Interestingly, the importance of timely sharing of surveillance data with all relevant stakeholders has also been recognized by other nations. For example, in Chile, a selection of key national and sub-national decision-makers were surveyed by the Influenza Surveillance Team at the MOH to evaluate how the influenza burden disease estimates and the surveillance data that underpins them were used to develop the national policies on seasonal and pandemic influenza. It was reported that timely reporting of surveillance and burden data and comprehensive communication to all stakeholders were two critical enablers of influenza policy development [6]. More user-friendly statistical reports and annual/semi-annual/routine fact sheets using infographics and user-friendly interactive websites are needed to expose all those interested to relevant data from influenza surveillance systems and other diseases. 

For COVID-19, simple statistical reports and infographics were an asset in communicating risk to the general public. These were available on daily basis and were effectively communicated in the daily press conferences set up by the MOH. Information presented included routinely collected data such as COVID-19-related testing, hospitalization, and deaths, which provided a clear message of the epidemic situation in Jordan to the general public and clearly corresponded with non-pharmaceutical intervention (NPI) measures. The available surveillance data were properly utilized for analyses, reporting, visualization, and dissemination in a regular and timely manner. 

### 3.7. Core Capability 7: National Respiratory Disease Surveillance and Reporting

Jordan has a well-established infrastructure for disease surveillance and a reporting structure that uses live (online) data entry. This reduces the time needed to conduct epidemiological investigations, allowing for swift decision making, with reporting of severe cases or clusters of unexplained respiratory diseases taking less than 48 h following detection. Furthermore, continuous training and educational activities have been comprehensive at the local and national levels to increase awareness regarding reporting of suspected events, rumors, or trigger events (e.g., clusters, unusual age distribution, or other changes in epidemiology). Scanning programs to monitor media and other informal data sources such as telephone hotlines and email services were established and routinely utilized to investigate rumors and suspected cases. Still, there seems to be a gap in the One Health reporting approach between participating ministries as direct communication channels are not established. This includes standardized definitions for what classifies as triggers and suspected events, which may affect the timeliness and accuracy of reports. There should be an agreement in place between ministries on what and when to report and at what level. This includes standardized case definitions for each zoonotic disease and disease-specific indicator(s) that trigger reporting and response within each ministry. 

During COVID-19, Jordan has expanded the audience of its surveillance reporting and awareness to include the general public and multiple media forms. It has engaged educational medical facilities and personnel to disseminate surveillance reports to inform the public about suspected or triggered events. As well, Jordan built onto the existing influenza surveillance system and gained access to timely reporting on not only confirmed COVID-19 cases but also suspected cases and contacts of confirmed cases. The focus is on human health with an aim to support online (live) reporting of zoonotic diseases of human significance within animals. The MOH hotlines and the very well-established NCSCM have also played a significant role in communicating with the general public and providing advice and support to suspected cases. This eliminated delays and reduced the exposure of the general public to health services when unnecessary. Notification systems between ministries, which were lacking for PIP, served as a hub to collect data and facilitate decision making using defined formal communication channels, standardized definitions for triggers and suspected events, and systematic notification systems that extended to the ministry of interiors and military personnel. The center proved able to facilitate access to critical information and eliminate routine bureaucracy while supporting information sharing with experts in the fields of virology and infectious diseases. 

### 3.8. Core Capability 8: Outbreak Response

An outbreak response system has been established in Jordan with well-trained personnel located within a centralized health system with limited decision-making power at the sub-national level. National experience has shown that central decision making allows for precise and timely decisions in PIPRP. 

Jordan’s outbreak response teams are multidisciplinary and include epidemiology, clinical assessment, specimen collection, and infection control experts. Good training within MOH provides sustainability for such teams. However, other functional expertise, including logistics, veterinary science, and environmental health sciences, is lacking and could add value to such teams on the ground. Moreover, the loss of trained staff within such teams due to movement centrally or within health districts and high staff turnover are of great concern. Logistical resources for response teams, including complete materials and equipment, are accessible and organized at both national and sub-national levels. A trained, equipped team begins responding to potential Public Health Emergency of International Concern (PHEIC) on-site within one day, submits laboratory specimens within 24 h, and subsequently provides preliminary results within 72 h of investigation. 

We observed that PIP infrastructure allowed for a coherent response to COVID-19 and decentralized decision making, especially in areas that were under regional lockdown. Human capital suffered significant fatigue with the closure of health facilities, and such demand for human capital was met by utilizing non-state actors for investigating cases and contacts. However, the shortage of essential medical equipment was also a problem in Jordan during the pandemic; this was a global phenomenon. 

### 3.9. Core Capability 9: Resources for Containment

The Jordanian MOH has focal points at the district level to allow for antivirals and personal protective equipment distribution to more than 75% of the country’s geographic area within 24 h. Antiviral prophylaxis is available for 20-day containment for a mid-scale containment effort for 12,000 persons (excluding staff involved in the outbreak response or containment effort). Government storage facilities of antivirals have adequate security, temperature control, inventory tracking, rotation, and stock refreshing. However, tabletop activities practicing the management and distribution of resources for containment have not been adequate; the MOH needs to conduct drills, simulations, or practices. Antiviral availability and utilization by healthcare professionals, first-line responders, and high-risk population subgroups is not well known in Jordan and needs to be explored. 

COVID-19 put unprecedented pressure on health systems with all activities being tested, including those related to mass containment. The utilization of the NCSCM has been an asset in guiding the proper utilization of available resources not related to health services. This permitted the effective distribution of materials within available storage facilities and allowed access to much-needed resources on time. 

### 3.10. Core Capability 10: Community-Based Interventions to Prevent the Spread of Influenza

Jordan’s PIP plan lacks clear instructions about how and when to restrict human movement or activities to control influenza spread, as well as community-based intervention plans to prevent the transmission of influenza at the sub-national level. Therefore, sub-national plans must be prepared and widely disseminated to the public in coordination with other ministries and should incorporate the One Health approach in terms of team building and sharing information. 

Cultural issues should be considered when designing NPI measures to combat the spread of infectious diseases, as social distancing plans and restrictions of population movements are limited by cultural barriers and economic ones. During the initial lockdown in Jordan, the national defense law was applied, and military control was activated to limit population movements within and between administrative boundaries. This was further facilitated by clear risk communication strategies utilizing the NCSCM. While prior to the pandemic, there was no clear pre-existing plan, significant measures were proposed based on the new epidemiologic situation, with most developing countries now having a plan to combat the spread should a new pandemic erupt. This is critical as, for example, clear guidance on regulating access to and maintenance of essential services (e.g., water supply, electricity, gas, trash collection, and grocery) during lockdowns. Such plans should be established, tested, disseminated, and regularly updated at the national and sub-national levels. 

### 3.11. Core Capability 11: Infection Control

PIP infection control was well established in Jordan prior to the start of the pandemic. Infection control standards exist for all levels of the healthcare system with a strategy for assessing quality assurance and compliance with follow-up in cases of non-adherence to standards. Regarding human capital, all staff members were trained in infection control practices, not just leadership or management personnel. Almost all hospitals at both national and sub-national levels had such training. Still, this should also be expanded to include all health facilities, including those unrelated to the MOH. Logistical resources for infection control, mainly infection control materials, are generally available at the health district level and comply with WHO practices, but they should also be available at all sub-national levels to ensure easy access and utilization during emergencies. Infection control committees are present at sub-national levels, including the hospital level, with established data collection and reporting practices. 

Infection control standards within each health facility should be up to the required standard during severe outbreaks or pandemics such as COVID-19. Jordan invested sufficient funding and political support into meeting this objective. This elevated infection control standards and ensured that access to logistics was efficiently coordinated. Jordan invested strongly in PIP, which allowed for implementing high standards when resources were available. The activation of infection control units within each healthcare facility was an asset that reflected properly on meeting the needs of COVID-19. 

### 3.12. Core Capability 12: Health Sector Pandemic Response

Health Sector Pandemic Response received the lowest score (0.8). Although clinical management guidelines for the care of patients with suspected/novel strains of influenza were developed and adequately disseminated, and staff members at sub-national levels were trained in them, human resources for health sector surge capacity were found to be limited, and thence, clear guidelines for increasing the human resources were recommended to meet future increases in influenza cases. Moreover, training volunteers and identifying retired professionals with appropriate skills could further bridge the gap in human capital surge capacity. In addition, physical facilities and equipment for the health sector surge capacity, including investments to increase the number of beds or home care services, which are essential during a pandemic to increase the availability of hospital beds, were limited. A formal plan to increase bed capacity should be established. Furthermore, limited planning for the care of the deceased during a potential pandemic was also noted. It was expected that an increase of suspected or reported influenza cases would generate a shortage of laboratory testing kits and increased demand for testing logistics and personnel. Epidemic appropriate action plan(s) were suggested for laboratory facilities to meet any surge in cases. 

During the COVID-19 pandemic, a lack of laboratory testing plans was evident when there was a sharp increase in new cases. The initial workload on laboratory services was met by equipment and personnel already within MOH capacity. However, when new COVID-19 cases began to increase, it was no longer the case. At that stage, private labs stepped up to meet the demand, and an electronic system was adopted to facilitate the handling of samples. Testing sites were also distributed geographically in Jordan to meet the national and sub-national needs. Still, the shortage of ventilators was evident, especially given the established demand prior to the pandemic. Regardless, Jordan has realized that Health Sector Pandemic Response was not as well established as other core capabilities. The establishment of mobile hospitals for COVID-19 patients was critical in Jordan but delayed until a later stage. Now, these hospitals could meet the demand of any surge in cases and better support the health system, allowing it to run smoothly while dealing with a sudden increase in demand for critical care services.

## 4. Discussion

Preparedness refers to ‘the capability of the public health and healthcare systems, communities, and individuals, to prevent, protect against, quickly respond to, and recover from health emergencies, particularly those whose scale, timing, or unpredictability threatens to overwhelm routine capabilities’ [15]. Therefore, evaluation of preparedness identifies not only immediate steps needed for influenza but also other epidemics of global interest. Overall, Jordan has a well-established Pandemic Influenza Preparedness and Response infrastructure that is positively reflected in its response to COVID-19. Surveillance, outbreak, and research activities performed well in the PIP risk assessment also reflected well on the COVID-19 response. Still, extra attention should be placed on social mobilization and health sector capacity building in terms of human capital and physical facilities and equipment. These reflections are valid for influenza and COVID-19 and could be proper for any potential pandemic. 

The results point out the need for continuous assessment of the PIP plan not only for influenza but also for any potential epidemic within the country or at regional or global levels. The way that the PIP reflected on COVID-19 preparedness has been previously suggested. The WHO 2020 PIP progress reported that countries with better PIP infrastructure were able to more efficiently utilize available PIP resources (surveillance, laboratory, outbreak management, risk communications, clinical surge capacity, and product deployment) to mitigate and control COVID-19. For example, multiple countries established PIP principles, systems, and capacities to fight against COVID-19 [6]. This highlights the collateral benefit of PIP for other emergencies, especially when considering that PIP may have been helpful in refining the COVID-19 response by helping to characterize key epidemiological features, understand the spread, severity, and spectrum of disease, identify the impact on communities and guide the use of countermeasures such as case isolation and contact tracing. While comparison of the PIP national assessment may not be feasible between countries within the Eastern Mediterranean region (given that PIP was only reported for Jordan), it is expected that the International Health Regulations (IHR) scores be comparable in terms of national preparedness and response to pandemics. If this holds true, then Jordan’s “developed capacity” against health security risks seems to be better than some countries within the region (Palestine, Syria, and Pakistan that were reported to be of IHR limited capacity score), yet similar to those for Lebanon, Morocco and Tunisia. Egypt, Oman, and other gulf states, however, were reported to have better capacity in this regard [6].

Investment in human capital in the fields of public health, disease investigation, surveillance, testing, as well as data analysis seems to be inevitable. Training personnel who could serve as a reserve for the first line of defense is also critical. Academic institutions could provide a valuable source of human capital ready to be deployed when needed. Utilizing the infrastructure within these institutions is also valuable when needed. Investment in laboratory equipment is crucial and could be improved using available resources within the private sector. 

In responding to an epidemic, the One Health approach should be a cornerstone in meeting the national and sub-national needs. Zoonotic diseases are a significant risk factor in Jordan, and there should be an investment to meet the risks associated with any new infectious disease. This includes expanding surveillance systems and ensuring proper integration between data systems for information dissemination to both governing bodies and the general public. This integration should also focus on animal and environmental health. 

Capacity building within the healthcare systems should focus on all stakeholders, not only those within the MOH. The private sector and non-state actors are essential players in developing health plans, especially during emergencies. Clear communication channels between major stakeholders should also be established appropriately. For example, plans and exercises should incorporate hospitals and laboratory services within the private sector. For Jordan, the utilization of the National Center for Security and Crises Management (NCSCM) has been an advantage as it was already established with trained personnel in crises management. The center was essential in providing crucial national data needed to effectively implement the closure of agencies and services (mitigation) when needed. The center also served as a hub where all relevant data regarding COVID-19 were collected and disseminated accordingly. The NCSCM may have served as a central unit representing the One Health approach within this context. For instance, the availability of virologists and infectious disease specialists from within the academic institutions made it easier to utilize such expertise in a timely manner and introduce a more multi-disciplinary approach to controlling the spread of COVID-19. 

Jordan’s properly established PIP outbreak response and surveillance systems were equipped to deal with the COVID-19 pandemic. The elasticity of moving some decision making to the sub-national levels and utilizing the NCSCM clearly facilitated a swift response and provided access to vital data needed to combat the spread of the disease. The political will and public trust in the health system facilitated proper risk communication with the public and the media. Building on the available wealth of experience in influenza allowed for more smart decision making related to COVID-19 and allowed the country to deal with potential risks associated with the disease properly. 

The current study is limited in that only one PIP assessment was conducted in 2018 as a risk assessment guide for Jordan. In addition, the inventory may not be entirely suitable as a guideline for the dissection of the response to COVID-19, but it allowed for some reflections on Jordan’s response utilizing available core epidemiological capabilities. COVID-19 vaccination was an essential component in the fight against the pandemic. Still, this indicator was not measured in the CDC inventory, and this is a major limitation that could affect the comparison of influenza plans to the COVID-19 pandemic. Future assessment tools should consider this indicator as a precise core capability and assess logistics associated with the development, availability, and distribution of the vaccine. PIP assessment tool focused mainly on human health and did not provide a One Health approach where all sectors are evaluated and assessed. This major limitation should be considered in future assessment and evaluation tools. 

## 5. Conclusions

In conclusion, Jordan’s experience with PIP may have served as a guide for establishing successful COVID-19 control and mitigation. The established PIP principles, systems, and capacities seem to have reflected well on fighting against COVID-19 in terms of more efficient utilization of available surveillance, laboratory, outbreak management, and risk communications. This reflection facilitated a better mitigation and control of COVID-19. 

## Figures and Tables

**Table 1:** Overview of 2018 Jordan’s twelve core capabilities of the CDC Pandemic Influenza Preparedness and Response Inventory.

Number	Core Capability	Result
1	Country Planning	Jordan’s Influenza Preparedness and Response Plan is a stand-alone document that comprehensively covers all aspects of preparedness and response. It has been properly disseminated with some evidence of testing. Coordination between relevant stakeholders is established with financial support available for the majority of the Plan. Jordan should further invest in updating the Plan and testing it routinely using standardized methods.
2	Research and Use of Findings for Pandemic Influenza Preparedness	Collaboration between human and animal health has been nationally established but is not completely active. Research priorities are available but not documented nor prioritized. Ministry of Health (MOH) staff are actively participating in research conferences and research activities and data has been utilized for decision making. Further utilization of surveillance data at the regional and international levels is of added value.
3	Communications	Pandemic influenza-related operational communications plan is established. Communication materials tailored for target audience need to be further developed and tested. Communication staff positions and channels could be developed/activated at the health district level.
4	Epidemiologic Capability	Adequate certified staffing for epidemiologic positions has been noted at the national level with adequate participation rate(s) for epidemiologists and public health professionals. In-house educational programs are well established and feed national and regional needs. Decision making is still centralized with limited abilities to make proper decisions at the local levels.
5	Laboratory Capability	Testing for influenza viruses is still centralized at the Ministry of Health Laboratory Directorate. Sub-national testing sites/capacities are not established. Laboratory SOPs need to be developed.
6	Routine Influenza Surveillance	A total of seven SARI and ILI sites, using standardized case definitions, produce nationally representative samples in Jordan. MOH publishes and disseminates epidemiologic and virology data regularly. Integrated surveillance system could be of added value to accurately capture SARI and ILI cases. Establishing a distribution list to share/publish data enhances decision making. Research agenda that utilizes an influenza surveillance system needs to be developed.
7	National Respiratory Disease Surveillance and Reporting	Awareness of the need to report suspected events is systematically established including those related to rumor reporting and media scanning. Cross-notification between Ministries of Health and Agriculture needs to be further developed for timeliness and quality of data.
8	Outbreak Response	Human resources for outbreak response are still centrally located and utilize comprehensive team members with available resources at the sub-national level. Staff turnover at the peripheral level is still an issue.
9	Resources for Containment	Adequate availability and storage of antivirals is noted but tabletop exercises and practices are needed. Utilization of antivirals by health professionals and at-risk groups needs to be investigated using research.
10	Community-Based Interventions to Prevent the Spread of Influenza	Social distancing strategies need documented plans or guidance that involve other stakeholders. Maintenance of essential services needs to be properly addressed and documented. Voluntary isolation and quarantine have been applied and are accepted by the community. Health-district level planning to prevent the spread of influenza needs to be addressed and documented.
11	Infection Control	A system to address infection control at all levels is established. Comprehensive participation in quality assurance is not documented and addressed. Human capital is adequately trained in infection control skills and standards. Infection control materials are generally available at the sub-national level. National plans to improve infection control should be established
12	Health Sector Pandemic Response	Human and physical facility and equipment surge capacities are limited. Plans to address human and physical capacities need to be developed, documented, and tested. While clinical management guidelines are established, guidelines to address care of the deceased need to be developed and addressed.

**Table 2:** Influenza core capability indicators’ matrix and scores, Jordan 2018.

Number	Core Capability Score	Indicator	Indicator Score (Level of Capability)
1	Country Planning = 2.0	Status of Plan	2
		Dissemination	2
		Exercises	2
		Coordination	2
		Resources	2
2	Research and Use of Findings for Pandemic Influenza Preparedness = 1.7	Collaboration	2
		Research Priorities	1
		Environment of Support	2
		Use of Data	2
3	Communications = 1.3	Status of Communications Plan	2
		Messaging	1
		Dissemination	1
		Staffing	1
4	Epidemiologic Capability = 2.3	Operational Status	2
		Epidemiologists	2
		Quality	2
		Training	3
5	Laboratory Capability = 2.0	Laboratory Network	1
		Bio-safety Level	2
		Methods	2
		Participation in WHO system	3
6	Routine Influenza Surveillance = 2.5	Integrated Surveillance	3
		Data Publication	2
		Timeliness	2
		Case Definitions	3
7	National Respiratory Disease Surveillance and Reporting = 2.0	Awareness of Need to Report	2
		Rumor Reporting	2
		Cross-notification	1
		Timeliness	3
8	Outbreak Response = 2.5	Human Resources	2
		Logistical Resources	3
		Exercises	2
		Activation of Team	3
9	Resources for Containment = 2.0	Availability of Antivirals	2
		Storage Facilities	2
		Exercises	1
		Distribution of Materials	3
10	Community-Based Interventions to Prevent the Spread of Influenza = 1.5	Social Distancing	1
		Critical Infrastructure	1
		Voluntary Isolation	3
		Percent of Districts with Plan	1
11	Infection Control = 2.3	Standards of Infection Control	2
		Human Resources	3
		Logistical Resources	2
		Institutionalization of Infection Control	2
12	Health Sector Pandemic Response = 0.8	Surge Capacity Human Resources	0
		Surge Capacity Facilities 1	0
		Surge Capacity Facilities 2	0
		Clinical Guidelines	3
		Surge Capacity Care of Deceased	1
Overall Core Capability Score (average) = 1.9			

## Data Availability

Some or all data generated during the study are proprietary or confidential in nature and may only be provided with restrictions. The datasets generated and analyzed during the current study are available with the corresponding authors.
